# Key factors in children’s competence to consent to clinical research

**DOI:** 10.1186/s12910-015-0066-0

**Published:** 2015-10-24

**Authors:** Irma M. Hein, Pieter W. Troost, Robert Lindeboom, Marc A. Benninga, C. Michel Zwaan, Johannes B. van Goudoever, Ramón JL Lindauer

**Affiliations:** Department of Child and Adolescent Psychiatry, Academic Medical Center, Meibergdreef 5, 1105 AZ Amsterdam, The Netherlands; Department of Clinical Methods and Public Health, Academic Medical Center, Meibergdreef 9, 1105 AZ Amsterdam, The Netherlands; Department of Pediatrics, Emma Children’s Hospital, Academic Medical Center, Meibergdreef 9, 1105 AZ Amsterdam, The Netherlands; Department of Pediatric Oncology, Erasmus Medical Center/Sophia Children’s Hospital, Dr. Molewaterplein 60, 3015 GJ Rotterdam, The Netherlands; Department of Pediatrics, VU University Medical Center, De Boelelaan 1117, 1081 HZ Amsterdam, The Netherlands

## Abstract

**Background:**

Although law is established on a strong presumption that persons younger than a certain age are not competent to consent, statutory age limits for asking children’s consent to clinical research differ widely internationally. From a clinical perspective, competence is assumed to involve many factors including the developmental stage, the influence of parents and peers, and life experience. We examined potential determining factors for children’s competence to consent to clinical research and to what extent they explain the variation in competence judgments.

**Methods:**

From January 1, 2012 through January 1, 2014, pediatric patients aged 6 to 18 years, eligible for clinical research studies were enrolled prospectively at various in- and outpatient pediatric departments. Children’s competence to consent was assessed by MacArthur Competence Assessment Tool for Clinical Research. Potential determining child variables included age, gender, intelligence, disease experience, ethnicity and socio-economic status (SES). We used logistic regression analysis and change in explained variance in competence judgments to quantify the contribution of a child variable to the total explained variance. Contextual factors included risk and complexity of the decision to participate, parental competence judgment and the child’s or parents decision to participate.

**Results:**

Out of 209 eligible patients, 161 were included (mean age, 10.6 years, 47.2 % male). Age, SES, intelligence, ethnicity, complexity, parental competence judgment and trial participation were univariately associated with competence (*P* < 0.05). Total explained variance in competence judgments was 71.5 %. Only age and intelligence significantly and independently explained the variance in competence judgments, explaining 56.6 % and 12.7 % of the total variance respectively. SES, male gender, disease experience and ethnicity each explained less than 1 % of the variance in competence judgments. Contextual factors together explained an extra 2.8 % (*P* > 0.05).

**Conclusions:**

Age is the factor that explaines most of to the variance in children’s competence to consent, followed by intelligence. Experience with disease did not affect competence in this study, nor did other variables.

**Clinical trial registration:**

Development and use of a standardized instrument for assessing children’s competence to consent in drug trials: Are legally established age limits valid?, NTR3918.

## Background

More pediatric drug trials are needed, however, historically the protection of children from research was often translated as simply excluding them from research [1]. Research with this vulnerable population involves unique ethical and legal considerations. Little is known about children’s capacities to meaningfully decide on research participation. Most laws present age limits for children to exercise their patient rights, however in pediatric practice age limits often do not reflect the ability of an individual child [2]. For clinicians and pediatric investigators it is critical to strike a proper balance in order both to protect children’s interests when they are not fully able to do so themselves and to respect their autonomy when they are.

Competence is task- and context specific [3], and a competent decision is required for a valid informed consent next to voluntariness and being well informed. Strictly speaking incompetence denotes a legal status that in principle should be determined by a court. However resorting to judicial review in every case of suspected incompetence would very heavily burden both the medical and legal systems, therefore there is good reason to continue the traditional practice of having clinicians determine patients’ competence [4]. In clinical practice competence is generally addressed as decision-making capacity [5], and in this article we use the terms interchangeably, referring to clinical assessment of capacity and not legal determination of competence [5].

Children are not accorded the presumption of competence in most jurisdictions. Although age is frequently assumed to be the best feasible parameter to assess children’s competence to consent, internationally the statutory age limits for asking children’s consent to research participation differ widely from 12 to 18 years of age [6]. In the Dutch case, regarding clinical research participation, a double informed consent (child and parent) is required for minors from the age of 12 until 18. Children younger than the set age limit are considered by definition incompetent to act for themselves, they can express affirmative agreement by giving assent [7]. Previous studies have shown that age is at best a proxy for developmental capacity [8–10] and other key determining factors are highlighted. Cognitive development, experience, dependence on parents, and peer influences are the child-specific factors described in previous work [2]. Additionally, the impact of the level of complexity and risk of the decision, ethnicity and socioeconomic status (SES) are mentioned in literature [2, 11] to be potential determining factors.

In our present study we examine which are key determining factors for children’s competence to give informed consent to clinical research.

## Methods

### Patient population

The details of the study participants and baseline characteristics are comprehensively described elsewhere [12]. Briefly, between January 1, 2012 and January 1, 2014 a cohort of 161 pediatric inpatients and outpatients between 6 and 18 years of age were enrolled, visiting hospitals in Amsterdam, Rotterdam and The Hague (the Netherlands). Inclusion criteria were eligibility for clinical research participation and speaking Dutch. The clinical research projects at offer were 10 randomized controlled trials and three observational studies at departments of allergology, oncology, pulmonology, ophthalmology and gastroenterology. The study protocol was approved by the institutional review boards at each site. Prior written informed consent was obtained from the parents or guardians of the children who served as participants, and of participants 12 years or older, and assent form participants under 12 years of age.

### Methods

Children’s competence was assessed by the MacArthur Competence Assessment Tool for Clinical Research (MacCAT-CR) interview. The MacCAT-CR guides clinicians and patients through the process of information disclosure required for informed consent, combined with an assessment of the patient’s capacities, in approximately fifteen to twenty minutes. The MacCAT-CR offers a structured overview of patients’ capacities on four subscales (understanding, appreciation, reasoning, expressing a choice) to base a competence judgment on. The Dutch version of MacCAT-CR was modified for children by using simple language to be understood by children of elementary school age, and adding questions on the influence of social relationships [2]. The version used will be available after arrangement of proprietary issues. We demonstrated that children’s competence to consent to clinical research can reliably and validly be assessed by using the MacCAT-CR [12].

A MacCAT-CR competent classification was considered present when at least two out of three of the experts rated the MacCAT-CR interview positive for competence, in other cases patients were classified incompetent. Additional patient data were collected, demographic characteristics included ethnicity. Number of trials previously participated in and duration of disease were measures used to express disease experience. Disease experience was arbitrarily categorized as low (no prior trial participation and duration of disease less than one month), moderate (no prior trial participation and duration of disease more or equal to one month) or high (prior trial experience). The level of education of the highest educated parent served as an indicator of SES, which we categorized: low (no primary school, primary school, special primary school, special secondary school); middle (preparatory secondary vocational education, secondary vocational education, senior general secondary school, preparatory scientific education); high (college, university). Complexity of the decision was categorized into subgroups by consensus between three researchers (LG, IH, PT): low (open trial or randomized trial without blinding), or high (randomized trial with either the use of placebo, or blinding, or both). Risk was categorized using the same manner: low (no risk), moderate (little risk) or high (possible risk). Cognitive capacities were expressed as intelligence quotient (IQ) and assessed by the Wechsler Nonverbal Scale of Ability short version (WNV). The WNV was administered by trained certified professionals (special education or psychology graduates) under supervision of a senior professional. Scores on the WNV could be categorized into three IQ categories: low (under 90), average (90–110) or high (110 or higher). Ethnicity was classified as Western European, Middle East, Surinam/Antillean or other.

Parent(s) were asked if they judged their child had the capacities to make a well-considered decision on giving informed consent, in other words, if they considered their child decision-making competent. We classified if the child decided to participate in the research project at offer or not, or if he/she had not decided yet.

### Data analysis

Effects of the child variables and contextual variables on a competence judgment were expressed with odds ratios (OR) and their 95 % confidence intervals obtained by simple logistic regression. ORs > 1 indicate higher odds of a competent judgment when a characteristic is present, ORs <1 a lower odds. We considered the following child variables that may “cause” a competent classification: age, gender, intelligence, disease experience, SES, ethnicity. Contextual variables considered were complexity, risk, parental competence judgment and decision to participate in a study. First, we entered all child variables simultaneously into a multiple logistic model to examine their association with a competent judgment as expressed by the Wald-test statistic and associated p-value. Then, we entered the child characteristics one by one into a new logistic regression model, the variable with the largest Wald statistic from the full model first, then the variable with the second largest Wald statistic and so on. To evaluate the independent contribution to the total explained variance in competent classifications for a child variable, we examined the increase in Nagelkerke R-square explained variance after entering a variable into the model. The influence of the contextual variables, was examined by the extra increase in R-square explained variance after adding them to the model that already included the child characteristics.

## Results

### Baseline characteristics

Characteristics of the study participants were described elsewhere [12], we will give a brief overview: of the 209 eligible children eligible for this study, 161 were enrolled, mean age 10.6 years (range, 6–18).

### Association of variables with competence

The distribution of the child and contextual variables are shown in Table 1. A higher age, higher SES, average or above average IQ, Western ethnicity, a less complex decision, a parental judgment of competence and a positive decision to participate in research were variables positively associated with competence to consent (*P* < 0.05).

**Table 1:** Distribution of variables among competent and incompetent children

	Total	Competent	Incompetent	Odds ratio	*P*
	(N = 161)	(n = 100)	(n = 61)	(95 % CI)	
Mean age in years (SD)	10.6 (2.8)	12.4 (2.4)	8.9 (1.6)	2.70 (1.96-3.71)^a^	<0.001
Male gender, N (%)	76 (47)	44 (44)	32 (53)	0.71 (0.38-1.35)	0.30
IQ, N (%)					
Low^b^	52 (32)	24 (24)	28 (46)	1.00	-
Average	66 (41)	44 (44)	22 (36)	2.33 (1.11-4.93)	0.03
High	43 (27)	32 (32)	11 (18)	3.39 (1.41 -8.15)	0.06
Disease experience, N (%)					
Low^b^	49 (30)	31 (31)	18 (30)	1.00	-
Medium	74 (46)	44 (44)	30 (49)	0.85 (0.41-1.80)	0.67
High	38 (24)	25 (25)	13 (21)	1.12 (0.46-2.71)	0.81
SES, N (%)					
Low^b^	18 (11)	6 (6)	12 (20)	1.00	
Middle	76 (47)	47 (47)	29 (48)	3.20 (1.10 - 9.6)	0.03
High	67 (42)	47 (47)	20 (33)	4.70 (1.55-14.3)	0.006
Ethnicity, N (%)					
Western European^c^	91 (56)	64 (64)	27 (44)	1.00	-
Other^a^	70 (44)	36 (36)	34 (56)	0.45 (0.23-0.86)	0.02
Complexity, N (%)					
Low^b^	36 (22)	31 (31)	5 (8)	1.00	-
High	125 (78)	69 (69)	56 (92)	0.12 (0.07-0.55)	0.001
Risk, N (%)					
Low/moderate^b^	145 (90)	88 (90)	55 (91)	1.00	-
High	16 (10)	10 (10)	6 (10)	1.02 (0.35 -2.96)	0.60
Parental competence judgment					
Incompetent^b^	34 (21)	7 (7)	27 (45)	1.00	-
Competent	125 (79)	92 (93)	33 (55)	10.8 (4.3-27.0)	<0.001
Decision to participate					
No	62 (39)	34 (34)	28 (47)	1.00	-
Yes	64 (40)	48 (48)	16 (27)	2.5 (1.2-5.3)	0.02
Do not know	34 (21)	18 (18)	16 (27)	0.9 (0.4-2.1)	0.86

^a^Odds ratio for a competent judgment per year older

^b^Reference category, parental judgment 2 missings, decision to participate 1 missing

^c^Other: Middle East (30 %), Surinam/Antilles (13 %) and “other”(1 %)

### Contribution of variables to competence

The contribution of each variable to the total explained variance in competent judgments is listed in Table 2. Age alone explained 56.4 % of the variance and IQ added another 12.7 % totaling 69.1 % explained by these two variables. SES (1.5 %), gender (0.5 %), disease experience (0.3 %) and ethnicity (0.1 %) together added another 2.4 % to a total of 71.5 % of the variance in competent judgments. The contextual variables (not in table) were all not significant and explained together less than 3 % extra in explained variance.

**Table 2:** Contribution of child variables to total explained variance in competence judgments

	Corrected Odds Ratio^a^	Wald *P*-value	Cumulative Naegelkerke R square
Age	3.95^b^	<0.001	56.4 %
IQ			69.1 %
Low^c^	1		
Average	6.80	0.007	
High	19.24	0.001	
SES			70.6 %
Low^c^	1		
Middle	2.03	0.41	
High	4.20	0.17	
Male	0.67	0.51	71.1 %
Disease experience			71.4 %
Low^c^	1		
Medium	1.51	0.52	
High	1.21	0.79	
Ethnicity			71.5 %
Western European^c^	1		
Other	0.57	0.69	

Contextual variables complexity, risk, parental judgment and decision to participate added another 2.8 % of the variance to a total of 74.3 % explained variance

^a^OR for a variable corrected for all other variables in the table

^b^Odds ratio for a competent judgment per year older

^c^Reference category

## Discussion

Results showed that age is the key factor that explains most of the variance in children’s competence to consent. IQ is the second important contributing factor. Other factors that could potentially make a causal contribution (gender, disease experience, SES, ethnicity) did not add significantly to the explained variance in competence judgments, nor did the contextual factors.

The high contribution of age for children’s competence to consent complements to recent findings on age limits [12]. Earlier work showed that competence to consent was unlikely in children younger than 9.6 years and in those older than 11.2 years, competence was probable [12]. These findings offer underpinnings for appropriate age limits in policies regarding children’s consent.

Cognitive development was described in theoretic literature to play a major role in children’s competence [10]. In our present study cognitive functioning expressed in an IQ explained a substantial part of the remaining variance in competent classifications after accounting for age. High intelligence in young children who are generally not accorded the presumption of competence may be a reason to doubt incompetence and require individual competence assessment, as well as low intelligence in children who are legally accorded competence to consent.

Against our expectations, experience with disease was not associated with competence in this study. Although some authors describe that children with personal experiences of illness can obtain greater insight and understanding than children of comparable age without these conditions [13], others argue that children with chronic medical disorders or life-threatening diseases might experience more difficulties in adaptation, social integration, treatment adherence, and development of autonomy than children without these conditions [14, 15]. One possible explanation of our findings is that these effects outweigh each other.

Complexity and risk of the decision did not demonstrate impact on competence judgments, although most authors agree that decisions concerning higher potential risk and more complexity frequently require a higher level of competence [13]. We consider that providing a clear explanation of the research procedures might counteract the impact of the level of risk and the complexity of the research on children’s competence. Or, the complexity and risks involved are intrinsically weighted with age and cognition of a child in a competence judgment. When corrected for these major contributing variables the influence of complexity and risk to competence should be negligible.

The finding that SES and ethnicity did not demonstrate associations with competence indicates that generalizability of competence studies in populations of different ethnicity and SES might be possible.

A positive parental competence judgment was associated with a 10-fold higher odds of a competence decision (Table 1), however parents judged their children more easily competent than the experts did. Our results show that parents express a high expectation regarding their children’s competence, allotting them more voice and responsibility than professionals would. Or articulated contrariwise, professional standards might be more precautionary than parents’ judgments in children’s competence assessment.

### Limitations

A limitation may be caused by the somewhat arbitrary decision to combine studies into low, middle or high classifications of complexity and risk. Unfortunately levels of risk and complexity are not yet well defined or quantifiable [16, 17]. The same limitation is valid for combining trial experience and duration of illness. Both familiarity with having a chronic disease as well as prior research participation are supposed to add to the child’s experience, but levels of experience are not well defined in literature either.

## Conclusion

The demonstrated major role of age explaining variance in children’s competence to consent to clinical research, together with the previously estimated age limits for children’s competence to consent, provide scientific underpinnings for proposals to modify the regulations regarding children’s consent. As age limits for asking children’s consent vary considerably between countries [6], possible practical implications of this study’s results that take into account legal, ethical, developmental and clinical perspectives need to be considered in that context. Advantages and drawbacks of standardized competence assessment in children on a case-by-case basis compared to application of a fixed age limit based on empirical evidence need further discussion (see also submitted manuscript, December 2014, IH, PT, GM, MdV, JvG, RJL). Furthermore, future research is needed to examine children’s competence to consent in the treatment context.

## References

[1] Knox CA, Burkhart PV (2007). Issues related to children participating in clinical research. J Pediatr Nurs.

[2] Hein IM, Troost PW, Lindeboom R, de Vries MC, Zwaan CM, Lindauer RJ (2012). Assessing children’s competence to consent in research by a standardized tool: a validity study. BMC Pediatr.

[3] Beauchamp TL, Childress JF (2008). Principles of Biomedical Ethics.

[4] Appelbaum PS, Grisso T (1988). Assessing patients’ capacities to consent to treatment. N Engl J Med.

[5] Appelbaum PS, Grisso T (2001). The MacArthur Competence Assessment Tool for Clinical Research (MacCAT-CR).

[6] Altavilla A, Manfredi C, Baiardi P, Dehlinger-Kremer M, Galletti P, Pozuelo AA (2011). Impact of the new european paediatric regulatory framework on ethics committees: overview and perspectives. Acta Paediatr.

[7] American Academy of Pediatrics Committee on Bioethics (1995). Informed consent, parental permission, and assent in pediatric practice. Pediatrics.

[8] Wendler DS (2006). Assent in paediatric research: theoretical and practical considerations. J Med Ethics.

[9] Martenson EK, Fagerskiold AM (2008). A review of children’s decision-making competence in health care. J Clin Nurs.

[10] Kendall PC, Suveg C (2008). Treatment outcome studies with children: principles of proper practice. Ethics Behav.

[11] Miller VA, Drotar D, Kodish E (2004). Children’s competence for assent and consent: a review of empirical findings. Ethics Behav.

[12] Hein IM, Troost PW, Lindeboom R, Benninga MA, Zwaan CM, van Goudoever JB (2014). Accuracy of the MacArthur Competence Assessment Tool for Clinical Research (MacCAT-CR) for measuring Children’s competence to consent to clinical research. JAMA Pediatr.

[13] Larcher V, Hutchinson A (2010). How should paediatricians assess Gillick competence?. Arch Dis Child.

[14] Luyckx K, Seiffge-Krenke I, Schwartz SJ, Goossens L, Weets I, Hendrieckx C (2008). Identity development, coping, and adjustment in emerging adults with a chronic illness: the sample case of type 1 diabetes. J Adolesc Health.

[15] Rassart J, Luyckx K, Goossens E, Apers S, Klimstra TA, Moons P (2013). Personality traits, quality of life and perceived health in adolescents with congenital heart disease. Psychol Health.

[16] Alderson P (2007). Competent children? Minors’ consent to health care treatment and research. Soc Sci Med.

[17] Scherer DG, Annett RD, Brody JL (2007). Ethical issues in adolescent and parent informed consent for pediatric asthma research participation. J Asthma.

